# Immunophysical analysis of corneal neovascularization: mechanistic insights and implications for pharmacotherapy

**DOI:** 10.1038/s41598-017-12533-x

**Published:** 2017-09-22

**Authors:** Youness Azimzade, Jiaxu Hong, Alireza Mashaghi

**Affiliations:** Leiden Academic Centre for Drug Research, Faculty of Mathematics and Natural Sciences, Leiden University, Leiden, The Netherlands

## Abstract

The cornea lacks adaptive immune cells and vasculature under healthy conditions, but is populated by both cell types under pathologic conditions and after transplantation. Here we propose an immunophysical approach to describe postoperative neovascularization in corneal grafts. We develop a simple dynamic model that captures not only the well-established interactions between innate immunity and vascular dynamics but also incorporates the contributions of adaptive immunity to vascular growth. We study how these interactions determine dynamic changes and steady states of the system as well as the clinical outcome, i.e. graft survival. The model allows us to systematically explore the impact of pharmacological inhibitors of vascular growth on the function and survival of transplanted corneas and search for the optimal time to initiatetherapy. Predictions from our models will help ongoing efforts to design therapeutic approaches to modulate alloimmunity and suppress allograft rejection.

## Introduction

Life of a multicellular organism is a relationship among cells and not a property of any one cell^1^. As such understanding the implications of inter-cellular interactions is crucial for understanding physiological dynamics of human organs, progression of diseases and response of our tissues to pharmacological interventions^2^. Pharmacological strategies developed for the manipulation of *in vitro* systems made of a single cell type commonly fail when applied *in vivo*, where other cell types contribute to the emergent response, even if these cells are not directly targeted^3,4^. Understanding cellular interactions is also important from an engineering perspective; organ-on-a-chip technologies are developing rapidly, and in contrast to initial efforts, more realistic organ-on-a-chip systems will have to include multiple cell types with potentially complex inter-cellular interactions^5,6^.

Damage to our tissues commonly leads to inflammation, a double-edged sword that may contribute to resolution of damage or to generation of additional damage^7^. Two types of vessels namely blood vessels and lymphatic vessels contribute to inflammatory processes by transporting immune cells into and out of the site of inflammation^8^. Vessels also bring nutrients, wash the waste, and modulate generation and resolution of edema (swelling due to excess of extra-cellular fluid)^7,9^. Immune cells, on the other hand, modulate the growth of blood and lymphatic vessels^10,11^ Resolution of inflammation and recovery of the tissue is ideally accompanied by regression of damage-induced neovessels and disappearance of the recruited immune cells^12,13^. In the case of excessive inflammation, the damage can persist or even increase with time. For example, when the cornea is subjected to sterile injury, inflammation emerges and then resolves. However, after corneal transplantation, the presence of the graft as a source of antigens can potentially lead to prolonged inflammation^14^.

**Figure 1:**
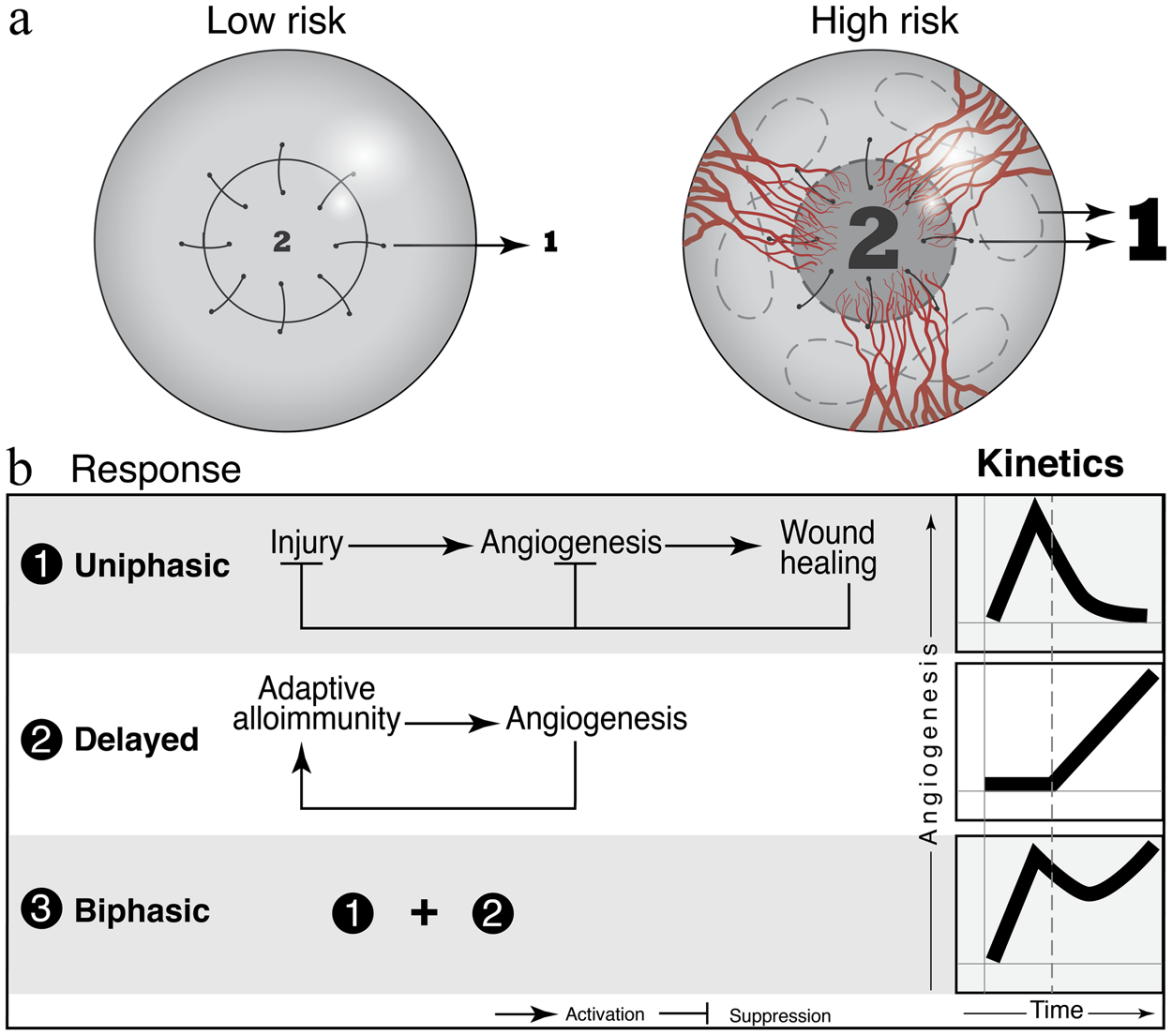
(**a**) Pre-existing vessels as the determinant factor for low risk and high risk setting for cornea. 1: the injury 2: immune response to the graft. The sizes of the numbers “1” and “2” in the figure indicate the intensities of the corresponding processes. (**b**) A simple model that explains the biphasic dynamics of angiogenesis as a combination of uniphasic dynamics (wound healing) and a delayed dynamics (adaptive immunity). Note that innate immune cells contribute to both uniphasic and delayed responses.

Here, we develop a simple and generic theoretical model of an integrated immune and angiogenic system and apply the model to understand rejection and tolerance of corneal transplants. Dynamics of the immune system have been subject to many previous studies^15–17^; vascular growth and regression has also been modeled previously^18–20^; yet the interactions between immunity and vasculature have not been thoroughly studied in the past and in particular the recently discovered contribution of adaptive immunity and associated inflammation to vascular dynamics^21^ has not been integrated into previous models. Corneal transplantation is a uniquely suitable system for such studies due to the inherent simplicity of the cornea, which lacks vessels and adaptive immune cells in healthy conditions. In our recent paper^14^, we have carefully measured vascular dynamics after corneal transplantation and identified non-monotonic changes in the amount of corneal vessels. Here, we take modeling approaches that incorporate the interactions between vessels and immune cells to develop a mechanistic understanding of the non-monotonic dynamics observed in various corneal graft settings. Our model not only considers the contributions of vessels to immune cell trafficking but also includes the contributions of vessels to water transport and generation versus resolution of edema. We study two clinically relevant settings^14^, (*a*) a low-risk setting in which a corneal graft is transplanted onto an avascular corneal bed, and (*b*) a high-risk setting in which a corneal graft is transplanted onto a previously vascularized cornea (Fig. 1a). Pre-existing vessels are known to increase the risk of corneal graft rejection by the immune system^22^. Despite its simplicity, our model generates dynamic features (Fig. 1b) similar to those we have recently described in an *in vivo* animal model^23^; thus, the model provides insights into possible underlying mechanisms that drive the complex dynamics. We then employ our model to address current problems in corneal pharmacology. In recent years, there have been seemingly contrasting reports that have suggested either lymphatic vessels^24^ or blood vessels^25^ as the ideal target for therapy. We try to resolve this controversy by studying two generic pharmacological manipulations of the system, namely suppression of blood vessels and suppression of lymph vessels, alone or in combination, and predict their impact on the functioning of the graft. Finally, we search for an optimal drug administration protocol that leads to the highest graft survival rate and fewest unwanted side effects.

## Model

The primary aim of developing the following model was to produce simulation results that qualitatively agree with experimental findings in murine models of corneal transplantation. This would allow us to develop mechanistic insights into the dynamics of the system. Where applicable, parameters were obtained from experimental data reported in the literature. Other parameters were estimated based on related observations in the literature and fitting values^23^ of variables to experimental values (For details, please see the Supplementary Information, Figs S1 and S2, and Tables I and II). The model developed herein can be used to generate hypotheses on optimal treatment and prevention of alloimmunity using specific and non-specific modulators of blood and lymphatic vessels.

A system of differential equations describing, in a simple way, the interactions between vessels, immune system, and the cornea was used to develop the proposed model. The proposed mathematical model is based on the following variables: the density of blood vessels (*V*_*B*_) which contains two components: *V*_*BN*_, density of blood neo-vessels; *V*_*BI*_, density of pre-existing blood vessels; *V*_*L*_, density of lymphatic vessels; *E*, the degree of edema; *T*, extent of trauma (wound); *D*, density of allo-sensitive immune cells and accompanying innate immune cells that together generate the delayed adaptive response. These variables are considered as dimensionless and are linked together with dimensionless coefficients. These coefficients were estimated by a combined method which incorporates a qualitative analogy with a least square method (see SI).

Assumptions related to the variables considered for developing the proposed mathematical model are described below. In allografts, evolution of *T* (trauma or tissue damage) depends (negatively) on the amount of available nutrients and (positively) on the trafficking of immune cells, both mediated by blood vessels. The suppression effect of blood vessels (*V*_*BN*_ + *V*_*BI*_; see below for explanation) on *T* is assumed to be linearly proportional to the value of *T* but it depends on the value of blood vessels nonlinearly, reaching a limit for large values of blood vessels density(*V*_*BN*_ + *V*_*BI*_). *D* which is promoted by vessels (see equations 4 and 5) has positive effect on the evolution of *T*. We shall represent these effects by a linear term:

1 $$\frac{dT(t)}{dt}=-{\alpha }_{{V}_{B}T}\frac{{V}_{B}(t)}{1+{V}_{B}(t)}T(t)+{\alpha }_{DT}D(t)$$

We categorize blood vessels into two groups. The first group is pre-existing vessels, *V*_*BI*_, which do not undergo temporal evolution but have blood vessel function such as carrying food and immune cells. The second group of blood vessels, *V*_*BN*_, is generated or suppressed in response to *T* and *D*. This group of vessels is also subject to spontaneous decay which is proportional to its value. Also, within a live organism, there is an upper limit for any variable due to space limits and other constraints. In the context of corneal transplantation, *V*_*B*_ could reach its maximum value, *V*_*Bmax*_, which we fix based on experimental results for blood vessels. We model these effects with linear terms:

2 $$\frac{d{V}_{BN}(t)}{dt}=-{\lambda }_{VB}{V}_{BN}(t)+[{\alpha }_{T{V}_{BN}}T(t)+{\alpha }_{D{V}_{BN}}D(t)]\,\mathrm{[1}-{V}_{B}(t)/{V}_{Bmax}]$$

*V*_*L*_ is assumed to behave similar to *V*_*BN*_ and thus its dynamics was modeled with similar equations but with distinct coefficients:

3 $$\frac{d{V}_{L}(t)}{dt}=-{\lambda }_{VL}{V}_{L}(t)+[{\alpha }_{T{V}_{L}}T(t)+{\alpha }_{D{V}_{L}}D(t)]\,\mathrm{[1}-{V}_{L}(t)/{V}_{Lmax}]$$

To ignite an adaptive immune response, alloantigens have to be transported by antigen presenting cells from graft site by *V*_*L*_ to the draining lymph node where adaptive immune cells (*D* cells) will be generated. *D*_*p*_ would be the indication of value of these intermediate steps (antigen presentation). *D*_*p*_ is promoted by *T* and *V*_*L*_ but because of the time-taking nature of these processes, *D*_*p*_ has time delay with respect to both *T* and *V*_*L*_. Finally *D*_*p*_ obeys spontaneous decay:

4 $$\frac{d{D}_{p}(t)}{dt}=-{\lambda }_{D}{D}_{p}(t)+{\alpha }_{T{V}_{L}{D}_{p}}T(t-{t}_{d})\,{V}_{L}(t-{t}_{d})$$

*D* is created from *Dp* and transported by *V*_*B*_ to the graft site and the adaptive immune cells at the graft site obey a spontaneous decay (equation 5)

5 $$\frac{dD(t)}{dt}=-{\lambda }_{D}D(t)+{\alpha }_{{V}_{B}{D}_{p}D}{D}_{p}(t)\,{V}_{B}(t)$$

Finally *E* gradually seeps into surrounding tissue, is removed by *V*_*L*_ from the graft and enhanced by *D* and *T*. All these processes are modeled in equation 6:

6 $$\frac{dE(t)}{dt}=-{\lambda }_{E}E(t)-{\alpha }_{{V}_{L}E}{V}_{L}(t)E(t)+[{\alpha }_{D{V}_{B}E}D(t)\,{V}_{B}(t)+{\alpha }_{TE}T(t)]\,\mathrm{[1}-E(t)/{E}_{max}].$$

## Results

**Figure 2:**
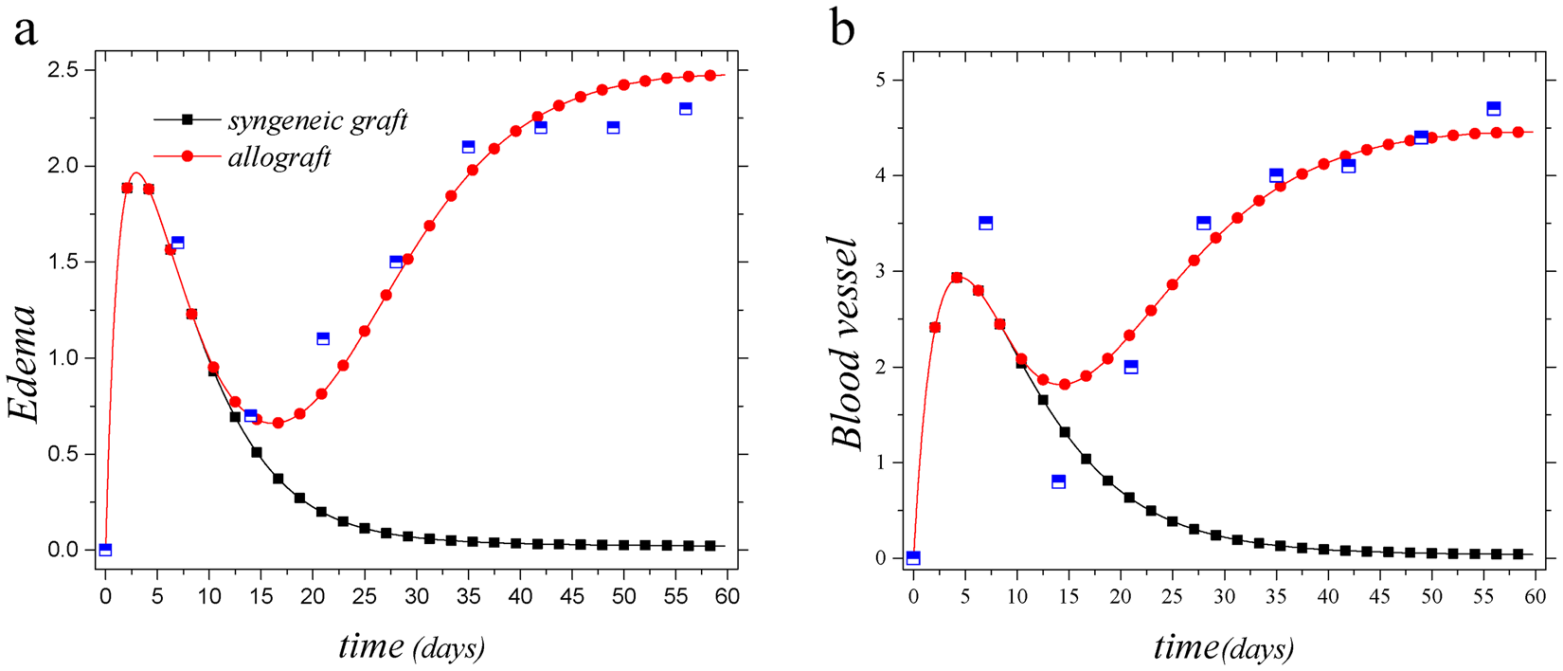
System dynamics in syngeneic graft, *t*_*d*_ = ∞ (uniphasic behavior) is compared to the dynamics of allograft (biphasic behavior), *t*_*d*_ = 7.4 *days* (see SI file for other coefficients). Blue half-squares present the experimental results (for dynamics of *T*, *D* and *V*_*L*_ see Figs S3a,b and S4 respectively).

The proposed set of equations simply model the major interactions that underlie inflammatory response to corneal allografts. This model implies that in the presence of adaptive immunity (ignited by the allograft), vessel growth follows a biphasic dynamics. Without consideration of adaptive immunity, our model predicts a uniphasic behavior which is compatible with a syngeneic graft or wound healing scenario (see Figs 1, 2, S3a,b and S4). If we eliminate adaptive immunity from our model, (simply by setting *t*_*d*_ = ∞), the system generates uniphasic dynamics irrespective of the magnitudes of the coefficients of the model.

**Figure 3:**
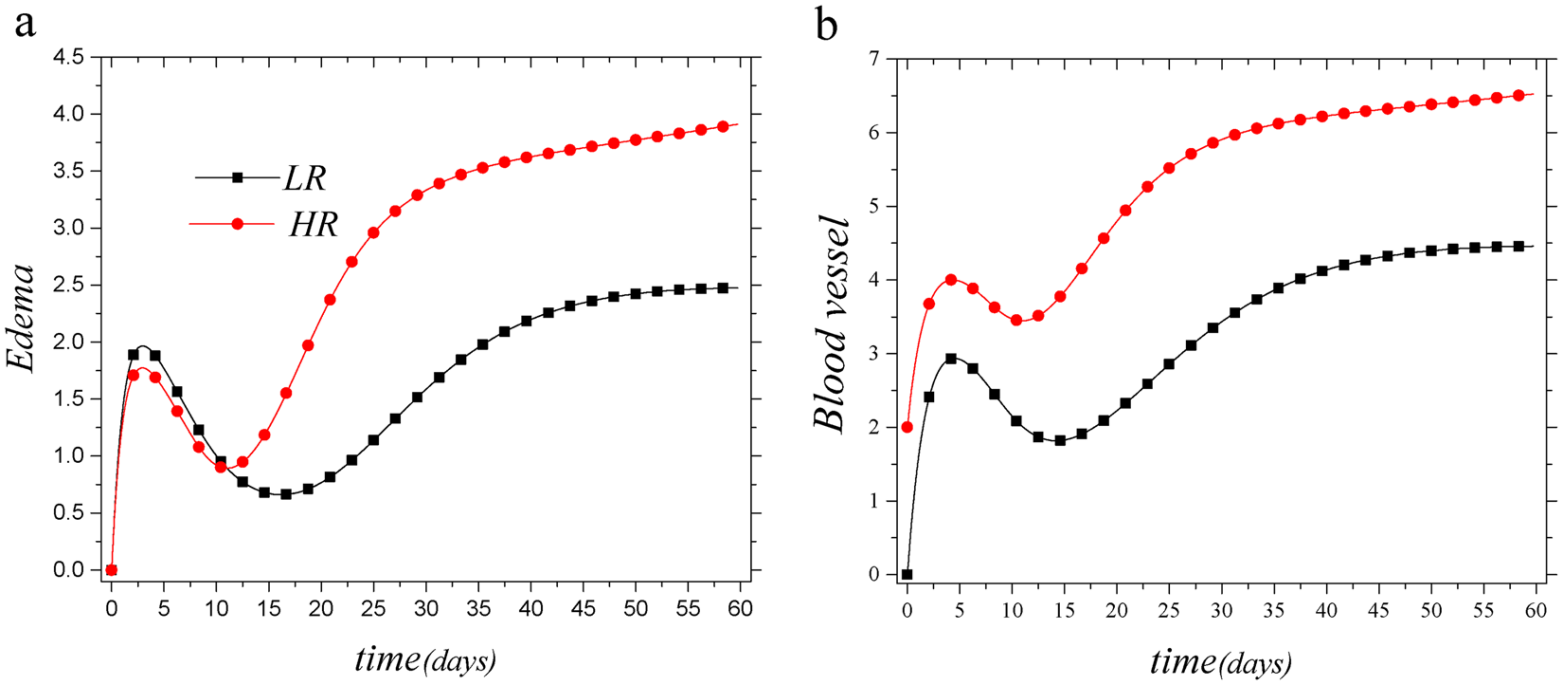
System response to vessels pre-existence as a risk factor for corneal transplantation. Two allograft conditions, i.e. LR (*V*_*B*_(0) = *V*_*BI*_ = *V*_*L*_(0) = 0) and HR (*V*_*B*_(0) = *V*_*BI*_ = 2, *V*_*L*_(0) = 1), are compared. In HR case, all variables will approach their upper limits at large time scales but for LR case, *V*_*L*_ takes small values at large time scales (with respect to *V*_*Lmax*_, see SI) as reported in experimental studies but *V*_*B*_ takes large value at large time scales (for dynamics of *T*, *D* and *V*_*L*_ see Figs S3c,d and S4 respectively).

Our model implies that pre-existing vessels in the host cornea increase the likelihood of graft failure; thus, the presence of the vessels is a risk factor for corneal graft failure. We probed this situation by changing the magnitude of pre-existing vascular system and assessing the risk of graft failure as indicated by loss of transparency (see Fig. 2). Function of cornea critically depends on its transparency. We find that systems with larger pre-existing blood vessels, *V*_*BI*_ (or *V*_*L*_(0)) have larger amounts of blood vessels, *V*_*B*_ (or *V*_*L*_) when evaluated several weeks post-transplantation. Higher amounts of blood and lymphatic vessels allow for higher antigen exposure and enhanced trafficking of immune cells. We assessed the amount of edema as well as the frequencies of inflammatory cells and found that our model predicts more intense edema and higher number of inflammatory cells within the graft, when recipient cornea is initially vascularized (see Figs 3, S3c,d and S4).

**Figure 4:**
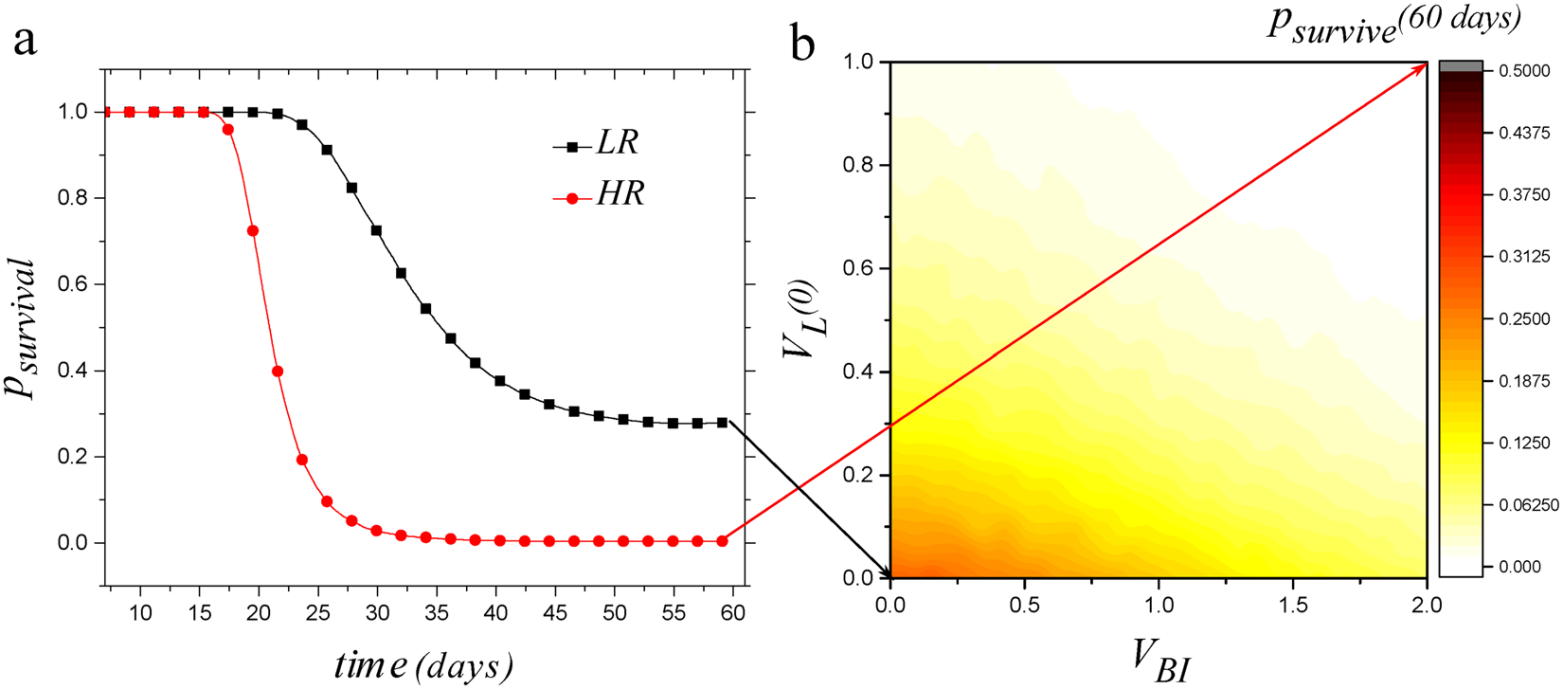
Graft survival probability for different allograft conditions. (**a**) Survival probability as a function of time for two states, *V*_*B*_(0) = *V*_*L*_(0) = *V*_*BI*_(1 + *r*) = 0, termed as LR, and *V*_*B*_(0) = *V*_*BI*_ = 2(1 + *r*), *V*_*L*_(0) = 1(1 + *r*), termed as HR in which *r* is a random number with normal distribution between [−0.5, 0.5]. *p*_*survival*_ equals to 0.29 and 0 for LR and HR respectively at t = 60 days. (**b**) Survival probability after 60 days as a function of initial amounts of vessels. From this figure one could find survival probability for any possible values of pre-existing vessels.

Next, we compared survival probability of grafts for recipients with and without pre-existing vessels. Consistent with the scoring system used in clinical settings^23^, we chose *E* = 2 as the critical value of edema: when *E* exceeded 2 we considered the graft as rejected (or failed). Thus, survival was defined for a system with *E* less than 2. To evaluate the system’s survival at a given time for different values of initial vessels, we changed *V*_*B*_(0) = 0 (respectively, *V*_*L*_(0) = 0) to *V*_*B*_(0) = *V*_*BI*_ × (1 + *r*) (respectively, *V*_*L*_(0) = *V*_*L*0_ × (1 + *r*)), where *V*_*BI*_ (*V*_*L*0_) is initial magnitude of blood (lymphatic) vessels and *r* is a random number with normal distribution between [−0.5, 0.5]. We also set *T*(0) = 1 + *r* in which *r* has the same range and distribution. We choose *r* from mentioned range for each one of 10000 iterations and calculated the dynamics of the system in order to have statistics on transplant survival for each magnitude of *V*_*BI*_ (*V*_*L*0_). The system response to different values of *V*_*L*0_ and *V*_*BI*_ has been investigated in Fig. 4.

**Figure 5:**
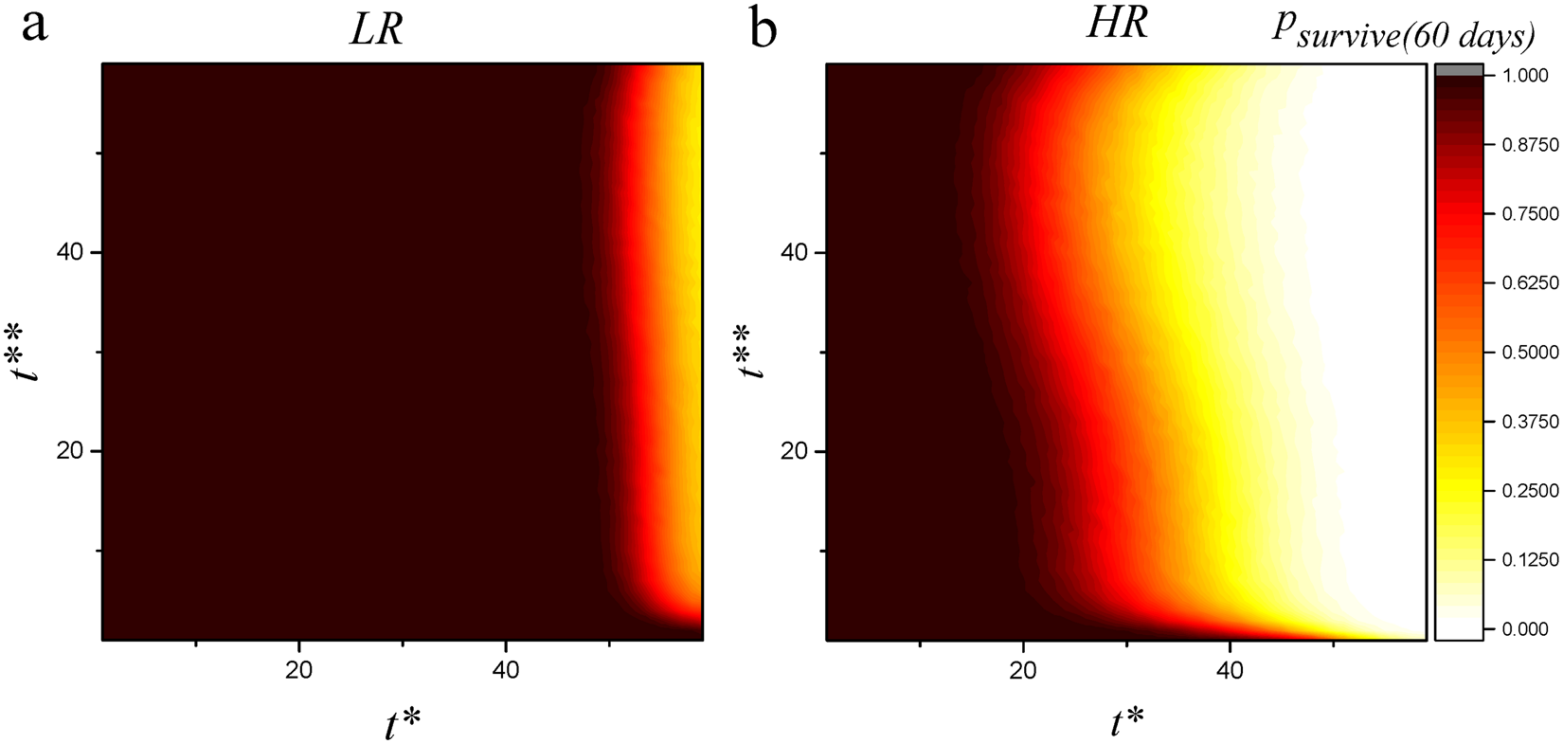
System response to specific therapy for high-risk (HR; *V*_*B*_(0) = 2, *V*_*L*_(0) = 1) and low-risk (LR, *V*_*B*_(0) = *V*_*L*_(0) = 0) conditions. After t* (t**) blood vessels (lymphatic vessels) were throughly suppressed. Based on these results, the optimum therapeutic approach entirely depends on vessels pre-existing values. For LR state which has *p*_*survive*_ = 0.29, suppression of either blood or lymphatic vessels increase *p*_*survive*_ to 1. In the case of HR, early suppression of blood vessels increases *p*_*survive*_ significantly, while suppression of lymphatic vessels does not change *p*_*survive*_. This result shows suppression of blood vessels as an appropriate strategy for increasing survival probability. In these cases we have *T*(0) = 1 + *r*.

In the remainder, we apply our model to predict the response of the system to pharmacological suppression of the blood and lymphatic vessels. There are several important open questions in the field: what is (are) the best target(s) for drug therapy for promoting allograft survival? Given a (specific or non-specific) drug, when is the optimal time to administer the drug? and what is the optimal protocol for drug therapy? The dynamic complexity of the *in vivo* system has made it difficult to answer these questions and it is timely to use modeling approaches to help designing drugs, animal experiments and clinical trials. Here, we suppose there is a drug which could specifically inhibit the growth of a certain kind of vessel (*specific targeting*). With such an assumption we define t* and t** which mark the timepoints when we start the administration of suppressors of blood and lymphatic vessels respectively. Upon administration of the drugs, for *t* > t*, equation 2 changes to $\frac{d{V}_{BN}(t)}{dt}=-{\lambda }_{V}{V}_{BN}(t)$ and for *t* > t**, equation 3 changes to $\frac{d{V}_{L}(t)}{dt}=-{\lambda }_{V}{V}_{L}(t)$. Both vessels start a spontaneous decay after therapy. We explored the space of t* and t** and assessed the outcome of the pharmacological interventions. The results are illustrated in Fig. 5.

Contrary to some studies which argued lymphatic vessels are the only possible target choice for suppression of graft rejection^24^, and in agreement with standard approach^25^, our model shows a significant increase in the acceptance probability of grafts with alternative strategies in which blood vessels are targeted. In the case of LR cases, suppression of the blood vessels could increase survival probability to 1 (Fig. 5 LR). In the case of HR, survival probability for early suppression of blood vessels reaches 1 (Fig. 5 HR). This result shows the crucial role of blood vessels compared to lymphatic vessels in rejection of corneal grafts.

Next, we suppose that the drug targets both vessel types but with distinct potency (*non*-*specific targeting*). We model this differential effect by introducing a coefficient, *P*. Equation 2 for *t* > *t*_*therapy*_ changes to

$$\frac{d{V}_{BN}(t)}{dt}=-{\lambda }_{V}{V}_{BN}(t)+P[{\alpha }_{T{V}_{BN}}T(t)+{\alpha }_{D{V}_{B}}D(t)]$$

Equation 3 also for *t* > *t*_*therapy*_ changes to

$$\frac{d{V}_{L}(t)}{dt}=-{\lambda }_{V}{V}_{L}(t)+\mathrm{(1}-P)\,[{\alpha }_{T{V}_{L}}T(t)+{\alpha }_{D{V}_{L}}D(t)]$$

**Figure 6:**
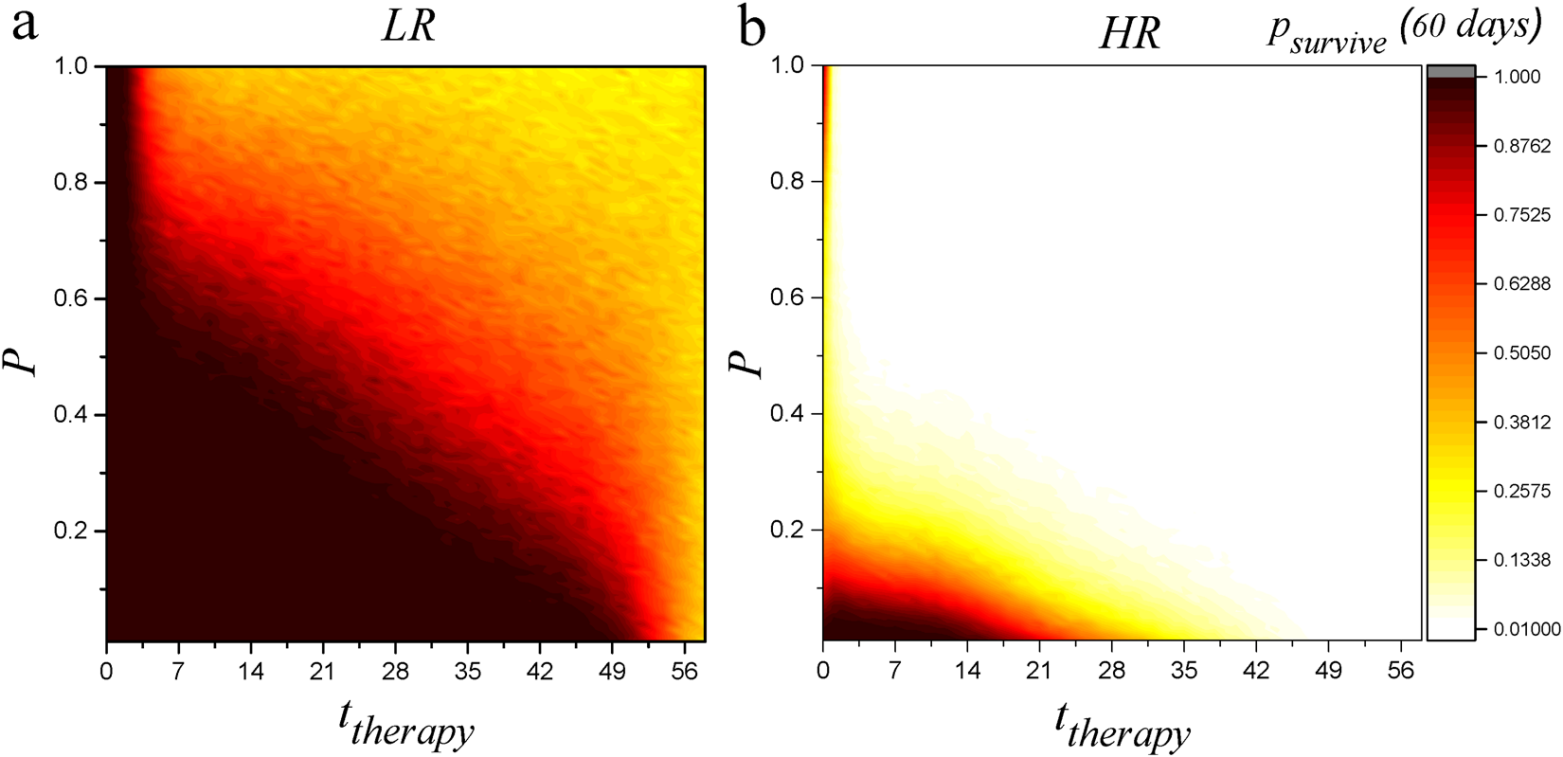
System response to non-specific therapy versus *t*_*therapy*_ (the time in which the administration of drug starts) and *P* in which blood vessel growth and lymphatic vessel growth are modulated by *P* and 1 − *P* respectively for HR (*V*_*BI*_ = 2 and *V*_*L*_(0) = 1) and LR (*V*_*BI*_ = 0 and *V*_*L*_(0) = 0) conditions. High values of *P* (or low values of 1 − *P*) indicate stronger suppression of lymphatic vessel growth or weaker suppression of blood vessel growth. For LR, early administration of suppressants of both kind of vessels would increase *p*_*survive*_ but for delayed therapies stronger suppression of blood vessels have better results in increasing *p*_*survive*_. In the case of HR situation, only early suppression of blood vessels will decrease rejection probability. In these cases, we have *T*(0) = 1 + *r*.

Based on proposed changes, obviously, the non-specific therapy approaches specific therapy when the drug has the highest potency for one type of vessels. In our model, large values of *P* (or low values of 1 − *P*) indicate high potency for suppression of lymphatic vessel growth and low potency for suppression of blood vessel growth. Figure 6 summarizes the outcome of non-specific therapy.

In the case of LR, early suppression of both kind of vessels, regardless of *P*, enhances survival probability to 1 but as the start-time of therapy increases, only lower values of *P* remain effective. This means that start time has an essential role in the efficiency of therapeutic approach and using anti-lymphatic/anti blood-vessel drugs which preferentially suppress blood vessels adds to effectiveness. In the case of HR, only lower values of *P* and short start-times are effective. Thus early administration of drugs which are more specific for suppression of blood vessels is the appropriate choice in this case (see Fig. 6 HR).

**Figure 7:**
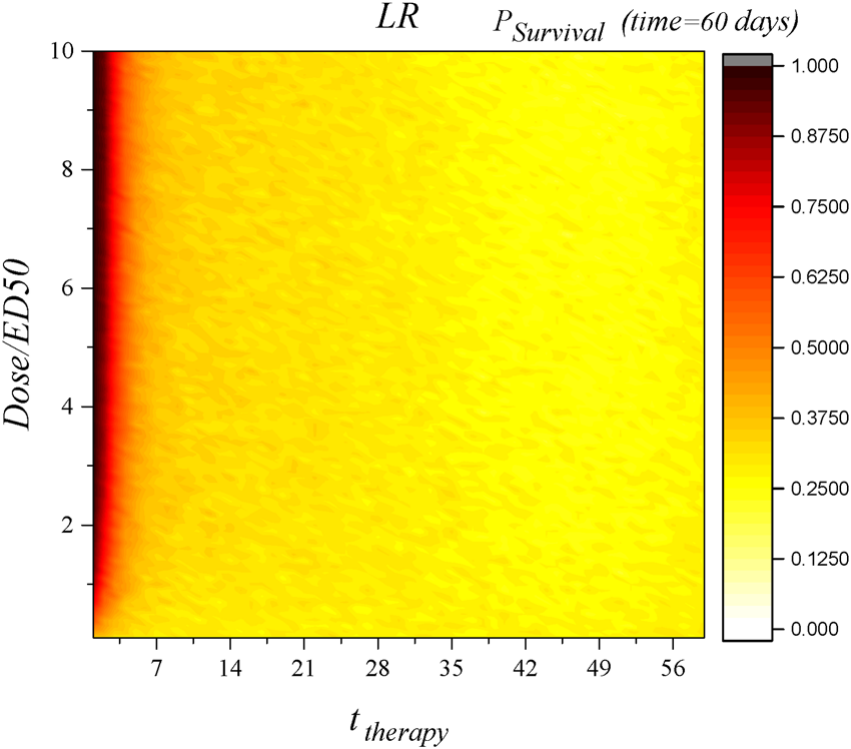
Partial inhibition (different values of *dose*) for *V*_*L*_ at LR. Early suppression of lymphatic vessels with *Dose*˜*ED*50 increase the survival probability to one and this is in agreement with complete inhibition method (for HR setting, we observed no positive outcome within the analyzed range of *Dose*; only very large values of *Dose* have positive effect on survival).

**Figure 8:**
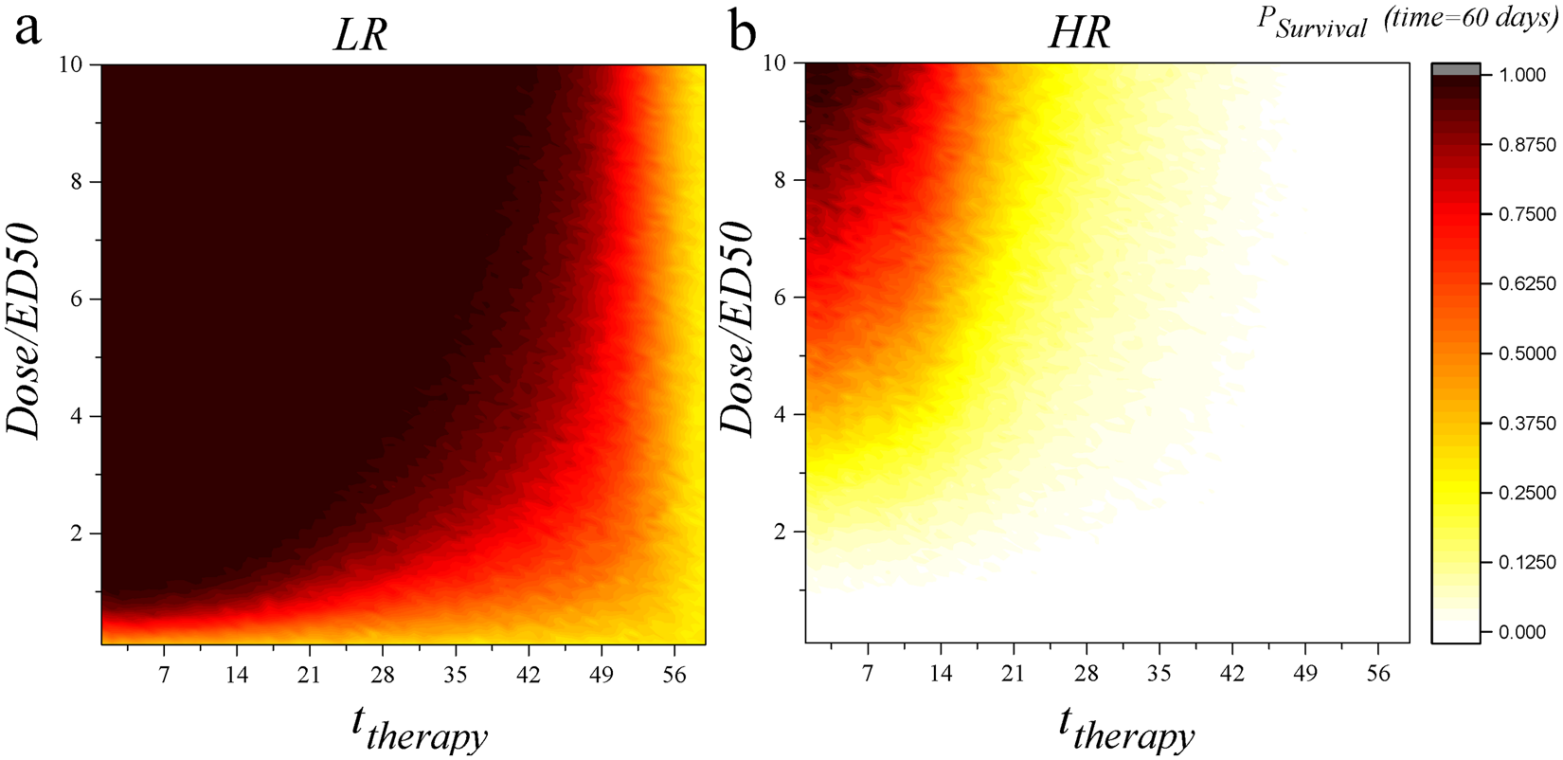
Response to different values of *dose* for *V*_*B*_ at LR and HR settings. For LR setting even small values of *Dose* at early times increase the acceptance probability of the graft. For HR setting higher values of *Dose* should be applied to increase survival probability.

Complete inhibition of vascular dynamics in our presented pharmacological analysis, despite being informative for drug development and providing mechanistic understanding, does not fully represent a realistic pharmacological manipulation scenario. In clinical settings, inhibitions are typically not complete but partial. As such, here we study partial inhibition and provide a more realistic model. Analogous to the non-specific approach, we define the value of *p* as the inhibition for lymphatic vessels. Inhibition (*I*) could be defined as: $I=\tfrac{Dose}{ED50+Dose}$ (consequently $Dose=\tfrac{IED50}{1-I}$) where *ED*50 is the dose that produces a quantal effect (all or nothing) in 50 percent of the population that takes it (median referring to the 50 percent population base). In each case, instead of considering *I* = 1 (as we have done previously and Fig. 5 contains results for such analysis) and changing the equation (here for *V*_*L*_) to: $\frac{d{V}_{L}(t)}{dt}=-{\lambda }_{V}{V}_{L}(t)$; inhibition was considered like below: $\frac{d{V}_{L}(t)}{dt}=-{\lambda }_{V}{V}_{L}(t)+\mathrm{(1}-I)$ $[{\alpha }_{T{V}_{L}}T(t)+{\alpha }_{D{V}_{L}}D(t)]$ or $\frac{d{V}_{L}(t)}{dt}=-{\lambda }_{V}{V}_{L}(t)+(1-\tfrac{Dose}{ED50+Dose})$ $[{\alpha }_{T{V}_{L}}T(t)+{\alpha }_{D{V}_{L}}D(t)]$. We also studied the partial inhibition of blood vessels as a therapeutic approach. Dynamics of blood vessels after applying intervention would be described as: $\frac{d{V}_{BN}(t)}{dt}=-{\lambda }_{V}{V}_{BN}(t)+\mathrm{(1}-I)$ $[{\alpha }_{T{V}_{BN}}T(t)+{\alpha }_{D{V}_{BN}}D(t)]$ or $\frac{d{V}_{BN}(t)}{dt}=-{\lambda }_{V}{V}_{BN}(t)+$ $(1-\tfrac{Dose}{ED50+Dose})$ $[{\alpha }_{T{V}_{BN}}T(t)+{\alpha }_{D{V}_{BN}}D(t)]$. As Figs 7 and 8 show, partial inhibition of blood vessels is more effective. Consequently, targeting blood vessels and suppressing them both partially and completely is more effective as compared to the suppression of lymphatic vessels.

**Figure 9:**
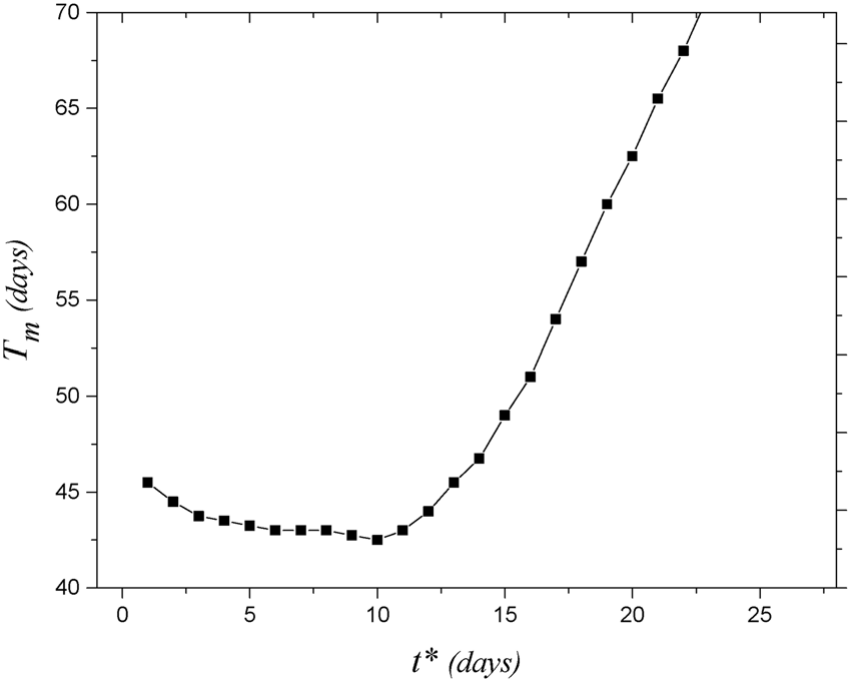
Minimum duration of blood vessel suppression, *T*_*m*_, with no build up of edema after cessation of drug theraoy versus the starting time of drug administration, t*, at HR setting. The minimum value of *T*_*m*_ which is equal to 42.5 *days* happens for t* = 10 *days*.

Since all drugs typically have some side effects, it would be of clinical value to find the shortest duration of therapy to minimize the side effects. Let’s focus on the HR setting and targeting of blood vessels. Duration of therapy is defined as a drug administration time interval which ensures that edema does not build up after cessation of therapy. For each starting time, we calculated the duration of therapy (for other assumptions see Figs S5 and S6). We estimated the minimum blood vessels suppression time, *T*_*m*_, during which the edema score drops to below two and we have no build-up of edema after cessation of therapy. A testable prediction that arises from this analysis is that the best time point for initiating the pharmacological suppression of blood vessels may be 10 *days* after transplantation (see Fig. 9).

## Discussion

Our study provides new insights into immunobiology of corneal transplantation and its clinical manifestations. Our model ascribed the bi-phasic behavior of the vessels to the presence of adaptive immunity. This behavior which has been recently observed in experiments^23^, as we showed, critically depends on the adaptive immune response. Besides, our model quantitatively showed the deteriorating effect of pre-existing vessels on graft survival. Despite its simplicity, our model successfully reproduced experimental survival rates reported for LR and HR grafts. We show how this model can be used to predict the outcome of pharmacological modulation of angiogenesis and lymphangiogenesis. For specific pharmacological targeting, suppressing blood vessels is effective. In the case of non-specific targeting, therapies that start early and suppress blood vessels more than lymphatic vessels are more effective because lymphatic vessels are responsible for extracting edema from tissue and when we suppress them, edema will be trapped at tissue and the risk of rejection would increase. On the other hand, blood vessels stimulate accumulation of edema at the graft site and when we suppress them, the accumulation of edema would be canceled. This result resolves existing controversy and shows the essential role of blood vessels in graft failure and challenges previous studies on the effect of lymphatic vessels on graft rejection^24^ Also, results confirm the experimental results which showed suppression of blood vessels to be more effective than suppression of lymphatic vessels^25^. In contrast to previous studies that have been focused on the contributions of blood or lymphatic vessels to immune cell trafficking, here we included the contribution of lymphatic vessels to resolution of edema, a process that critically affects the clinical status of the graft. As such, our study provides new insights by analyzing the tradeoff between immune cell trafficking and dynamics of edema when lymphatic vessels are modulated pharmacologically. Our study provides a guideline for researchers working on developing anti-angiogenic drugs and for clinicians who wish to design therapeutic strategies for patients undergoing corneal transplantation. Predictions from our model, even if they might not be intuitively unexpected in certain situations, could provide a rational for these researchers that otherwise have to perform blind screening. The latter is a major obstacle considering that drug development and clinical trials are costly.

Finally, our paper is, to our knowledge, the first to bridge the fields of theoretical systems biophysics and cornea research. As such we hope this study will ignite interest among ophthalmologists to consider the benefits of systems biophysics approaches to clinical problems. It should be noted that the mathematical model described here is clearly a simplification of the complex process of corneal transplantation. For example, it lumps all the adaptive immune response which involves contributions from T cells, macrophages, dendritic cells, etc. into one species, introduces functions whose precise form is unknown, and describes the formation of new blood vessels in terms of (cell) densities. Opacity is assumed to be caused primarily by edema and partly by infiltration of inflammatory cells; on long time scales scarring may also occur and this has not been included in our model. Nonetheless, the proposed mathematical model represents a starting point in the modeling of one aspect of corneal transplantation, that is, the effect of lymphatic and blood vessels on graft survival. Our efforts here were directed at focusing on multiple well established links between immunity and angiogenesis. The list of interactions considered herein clearly has room for expansion as relevant biological complexities continue to be experimentally unveiled. This work provides a mathematical model that enables simulations of how therapeutic modulation of blood and lymphatic vessels may influence transplantation outcomes. The simulated results agree with a variety of previously described experimental findings. As such, the model may represent a useful tool for analyzing strategies for improved graft survival and generate hypotheses for biological testing. Importantly, the current model lends itself to refinement, as future versions will be able to address additional factors implicated in the systems biology of the cornea.

## Electronic supplementary material

Supplementary information

## References

[1] Russell, P., Hertz, P. & McMillan, B. *Biology*: *The Dynamic Science*, https://books.google.com/books?id=bLgaCgAAQBAJ (Cengage Learning, 2016).

[2] Griffith LG, Swartz MA (2006). Capturing complex 3d tissue physiology *in vitro*. Nat. reviews Mol. cell biology.

[3] Esch EW, Bahinski A, Huh D (2015). Organs-on-chips at the frontiers of drug discovery. Nat. reviews Drug discovery.

[4] Neuži P, Giselbrecht S, Länge K, Huang TJ, Manz A (2012). Revisiting lab-on-a-chip technology for drug discovery. Nat. reviews Drug discovery.

[5] van Duinen V, Trietsch SJ, Joore J, Vulto P, Hankemeier T (2015). Microfluidic 3d cell culture: from tools to tissue models. Curr. opinion biotechnology.

[6] Zheng, F. *et al*. Organ-on-a-chip systems: Microengineering to biomimic living systems. *Small* (2016). 10.1002/smll.201503208 26901595

[7] Chung AS, Lee J, Ferrara N (2010). Targeting the tumour vasculature: insights from physiological angiogenesis. Nat. Rev. Cancer.

[8] Abbas, A. K., Lichtman, A. H. & Pillai, S. *Cellular and molecular immunology* (Elsevier Health Sciences, 2014).

[9] Adams RH, Alitalo K (2007). Molecular regulation of angiogenesis and lymphangiogenesis. Nat. reviews Mol. cell biology.

[10] Crowther M, Brown N, Bishop E, Lewis C (2001). Microenvironmental influence on macrophage regulation of angiogenesis in wounds and malignant tumors. J. Leukoc. Biol..

[11] Bruno A (2014). Orchestration of angiogenesis by immune cells. Front. oncology.

[12] Gurtner GC, Werner S, Barrandon Y, Longaker MT (2008). Wound repair and regeneration. Nat..

[13] Eming, S. A., Martin, P. & Tomic-Canic, M. Wound repair and regeneration: mechanisms, signaling, and translation. *Sci*. *translational medicine* **6**, 265sr6–265sr6 (2014). 10.1126/scitranslmed.3009337 PMC4973620 25473038

[14] Inomata, T., Mashaghi, A., Di Zazzo, A. & Dana, R. Ocular surgical models for immune and angiogenic responses. *J*. *biological methods* **2** (2015). 10.14440/jbm.2015.78 PMC4636218 26550579

[15] Molina-Pars, C. & Lythe, G. *Mathematical models and immune cell biology* (Springer Science & Business Media, 2011).

[16] Eladdadi, A., Kim, P. & Mallet, D. *Mathematical Models of Tumor*-*Immune System Dynamics* (Springer, 2014).

[17] Marchuk, G. I. *Mathematical modelling of immune response in infectious diseases*, vol. 395 (Springer Science & Business Media, 2013).

[18] Peirce SM (2008). Computational and mathematical modeling of angiogenesis. Microcirc..

[19] Qutub AA, Mac Gabhann F, Karagiannis ED, Vempati P, Popel AS (2009). Multiscale models of angiogenesis. IEEE Eng. Medicine Biol. Mag..

[20] Spill F, Guerrero P, Alarcon T, Maini PK, Byrne HM (2015). Mesoscopic and continuum modelling of angiogenesis. J. mathematical biology.

[21] Di Zazzo A (2016). Proangiogenic function of t cells in corneal transplantation. Transplantation.

[22] Hjortdal, J. *Corneal Transplantation*, https://books.google.nl/books?id=J9HjCgAAQBAJ (Springer International Publishing, 2015).

[23] Inomata T (2017). Kinetics of angiogenic responses in corneal transplantation. Cornea.

[24] Dietrich T (2010). Cutting edge: lymphatic vessels, not blood vessels, primarily mediate immune rejections after transplantation. The journal of immunology.

[25] Dohlman TH (2015). Vegf-trap aflibercept significantly improves long-term graft survival in high-risk corneal transplantation. Transplantation.

