# Supplementary material for: Immunophysical analysis of corneal neovascularization: mechanistic insights and implications for pharmacotherapy
Source: Sci Rep. 2017 Sep 22;7:12220. doi: 10.1038/s41598-017-12533-x (PMC5610330; doi:10.1038/s41598-017-12533-x)
Supplement: Supplementary file 1 — Supplementary information [file 41598_2017_12533_MOESM1_ESM.pdf]

SUPPLEMENTARY INFORMATION
Immunophysical analysis of corneal neovascularization: mechanistic insights and implications for pharmacotherapy

Youness Azimzade,[1] Jiaxu Hong,[1] and Alireza Mashaghi[1,*]
[1]*Leiden Academic Centre for Drug Research, Faculty of Mathematics and Natural Sciences, Leiden University, Leiden, The Netherlands*

NUMERICAL SOLUTION OF DIFFERENTIAL EQUATIONS

Equations were solved numerically using the Euler method. We used the following process to solve the equations: $X(t+\triangle t) = X(t) + \frac{dX(t)}{dt}\triangle t$ where $\triangle t$ is the time step of the model and is set to 10 minutes. Lower values of $\triangle t$ generate the same results. For each case, $\frac{dX(t)}{dt}$ was derived based on known interactions between model parameters.

TERMINOLOGY

We used the following convention for naming coefficients: for direct stimulation or suppression effect, we use $\alpha_{X_1X_2}$ in which X_1 is the actor parameter and X_2 is the target parameter. For example, α_{V_BT} is the contribution of blood vessels to trauma healing. If more than one parameter contributes to an action on another parameter, the corresponding coefficient contains all those parameters as actor parameters. Furthermore, for spontaneous decay, we used λ_X for corresponding decay rate. Table II lists the exact definitions for all coefficients.

COEFFICIENT ESTIMATION

In order to estimate coefficients, we used a two-step process. First, we estimated the value of coefficients based on qualitative arguments and by generating results that agree closely with the experimental data. Then we used a quantitative approach to further improve our estimations.

Qualitative Estimation

To estimate each coefficient, we need to understand its contribution to the dynamics of the model and to compare it with the data from the dynamics of the real system [1]. Some coefficients were directly estimated and some others were obtained through screening reasonable ranges as follows: Figure 1B in [1] shows that value of V_B for HR setting approaches 8; thus, we set $V_{Bmax} = 8$. Besides, Figure 1C in [1] shows saturation of E in the case of HR at long time scales. Accordingly, the upper limit for E is close to 6. Simply by comparing experimental measurements for V_B and V_L (Figures 2 and 3 [1]) we can roughly estimate $V_{Lmax} = V_{Bmax}/2 = 4$. Adaptive immune response comes with a delay with respect to other components of our model. Consequently, we can ignore terms related to adaptive immunity for early times. As such, T only depends on V_B (for $t < 14$ *days*). Figure 1B in [1] shows that for the LR case, the value of V_B starts to fall at $t = 7$ *days*. This means that the value of T has changed significantly and reaches a low level at this time. Value of $\frac{V_B(t)}{1+V_B(t)}$ during the first 7 days is roughly equal to 0.4 (based on simply averaging on this function for $t = 0$ and $t = 7$ and available data for V_B). We suppose that the value of T reaches 0.2 (recall $T(0) = 1$). By inserting all estimated values to equation 1 the modified version, $\frac{dT(t)}{dt} = -0.4\alpha_{V_BT}T(t)$, is easy to solve for the boundary conditions ($T(0) = 1$,$T(7) = 0.2$) and $\alpha_{V_BT} \approx 0.5$. On the other hand, we know that V_B has a maximum at $t = 7$ *days*; thus, we have $\frac{dV_{BN}(t)}{dt}|_{t=7} = 0$. This relation leads to $\lambda_{VB} = \alpha_{TV_{BN}}/35$. For $0 < t < 7$ we can assume $T_{avarage} = 0.6$ and again $V_{Baverage} = 1.8$. The modified version of equation 2, would be $\frac{dV_{BN}(t)}{dt} = \frac{V_{BN}(7)-V_{BN}(0)}{7} = 0.5\alpha_{TV_{BN}}$. As a result, we have $\alpha_{TV_{BN}} = 1$, $\lambda_{VB} = 0.03$.

V_L in the LR case and at the early times has similar dynamics (equation 3). Value of V_L in this regime is about half of the value of V_B [1](Figures 2B and 3B). Therefore, we can argue for $\alpha_{TV_L} = \alpha_{TV_{BN}}/2$. Besides, we know

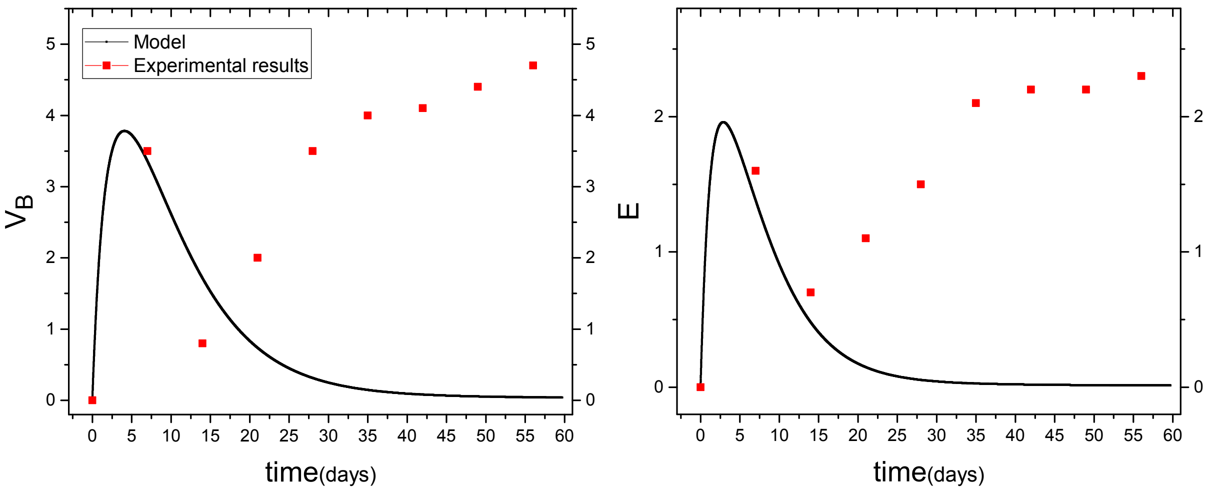

FIG. 1: Experimental results [1] is compared to the results of our proposed model for early times, $t < 14$ *days*

that the value of V_B declines for LR. For large times, spontaneous decay is the only weakening term in the evolution of V_B, then λ_{VL} is the only responsible coefficient for this behavior and from results for long time and we found $\lambda_{VL} = 4\lambda_{VB}$.

Equation 6 in early times reduces to $\frac{dE(t)}{dt} = -\lambda_E E(t) - \alpha_{V_L E} V_L(t)E(t) + \alpha_{TE}T(t)[1 - E(t)/E_{max}]$. Average value of E in this range is equal to 0.85. Figure 1C in [1] shows that the behavior of E is analogous to the behavior of V_B with half value. Here we use our estimations in previous parts to estimate values of the remaining coefficients. This analogy implies $0.85\alpha_{V_L E} + \lambda_E = \lambda_{VB}$ and $\alpha_{TE} = \alpha_{TV_{BN}}/2$.

After successfully creating the experimental results for early times, we consider longer time periods ($t > 14$ *days*). The rise of parameters after 14 *days* implies that the adaptive immune response has come to play prior to this time point. To see such behavior we have to set $t_d < 14$ and it turns out that $t_d = 7$ *days* fits best with experimental results. At long time scales, we know that V_L reaches zero in the case of LR setting. As a result, λ_E is the only coefficient with weakening effect on the dynamics of E and we found its value by monitoring the behavior of E in long time periods.

An accurate value of D is not available, however, we can come up with an estimate. The steady dynamics of other parameters in the long term suggest a steady dynamics for D itself. Thus, we expect a rise after a time delay and rather a constant value for D then. Therefore, D_P should be set to satisfy this situation. Similar logical arguments can be made for D_P. Its value should start to rise after $t = t_d$ and have smooth behavior in long times. We set coefficients in order to generate the mentioned behavior for D_P. This rise and subsequent smooth behavior of D_P leads to an initial rise and a subsequent steady dynamics in long time periods for D. Finally, experimental results were reproduced by tuning relevant parameters, while keeping the steady dynamics of D and D_P invariant (see Figure S3). Again, we stress that our procedure involved an initial estimation which was subsequently tuned to reach the best fits with the experimental results. Figure S2 shows the qualitative agreement between our estimation and available experimental results.

Quantitative Refinement

The above mentioned qualitative estimations of the coefficients were conducted to find a reasonable domain for each coefficient, and for a more accurate fit we did a quantitative approach as we describe below: we consider three main parameters from LR model, i.e., evolution of V_B, V_L and E as our baseline experimental data. V_B and E have been clinically measured but there exist no equivalent experimental data for V_L. By comparing Figure 2B and Figure 3B in [1] we learn that $\frac{V_B}{V_L} > 2$ for $t < 28$ *days* (for HR this ratio is different). Besides, for larger times, the value of V_L decays (Figure S4). As a result, we consider Table I data as our reference for fitting.

To obtain the best fit, we use the least square method and calculate $R = \sum_i \sum_{t=0}^{56} (X_i(t)_{sim} - X_i(t)_{exp})^2$ with $X_i = V_B, V_L$ and E for LR. Extreme minimum value of R as the best fit is obtainable by screening the space of coefficients and it turns out to be equal to 2.9 for the coefficient values listed in Table II. We note that these values

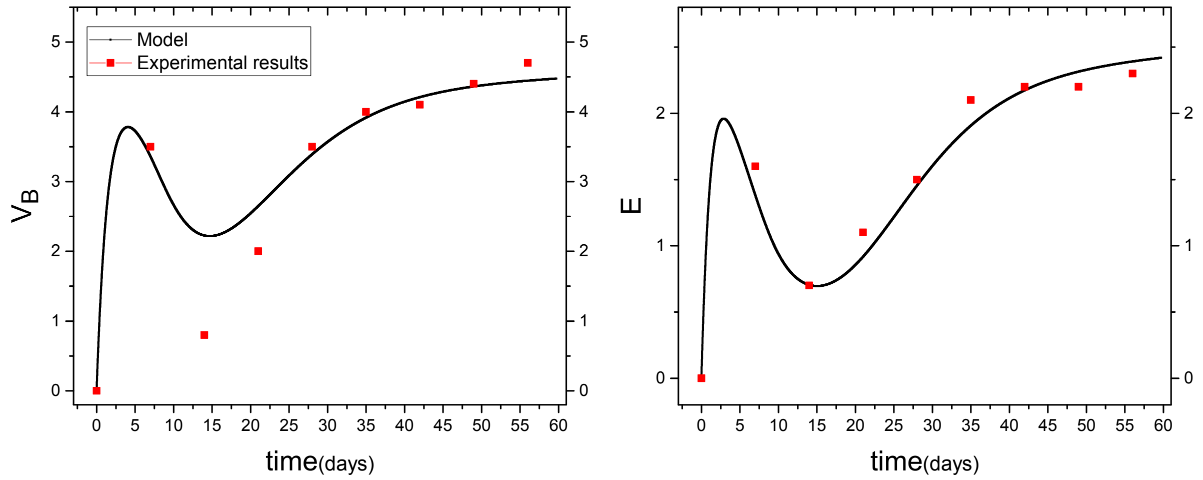

FIG. 2: Experimental results [1] compared with the results of our proposed model after qualitative fitting of the model parameters.

TABLE I: Experimental data used for quantitative method.

Time ($days$)	V_B	E	V_L
0	0	0	0
7	3.5	1	1.6
14	0.8	0.7	0.3
21	2	1.1	0
28	3.5	1.5	0
35	4	2.1	0
42	4.1	2.2	0
49	4.4	2.2	0
56	4.7	2.3	0

properly reproduce the behavior of HR setting as well.

SENSITIVITY ANALYSIS AND CONFIDENCE REGION

Here we perform sensitivity analysis to check the dependency of the model on our estimated parameter values. In other words, we ask how much deviation from each estimated value is needed to generate significant alteration in the dynamics of the model. We define a confidence region [2] around each coefficient value where the original dynamics of the model is conserved despite the introduced perturbation. To define the region, we consider R, as defined below, as the identification factor: R ($R = \sum_i \sum_{t=0}^{56} (X_i(t)_{sim} - X_i(t)_{exp})^2$ with $X_i = V_B, V_L$ and E for LR). We tune each coefficient until the value of R exceeds 4. Through this procedure we do a sensitivity analysis and obtain a confidence region for each coefficient (see Table II).

SHORTEST DURATION OF THERAPY

Since drugs typically carry some side effects, it is of high clinical value to find the shortest duration of therapy to minimize the side effects. To find the duration of therapy, we measured the time between the start of drug administration and the time when the edema score drops to below two. For both specific and non-specific approaches,

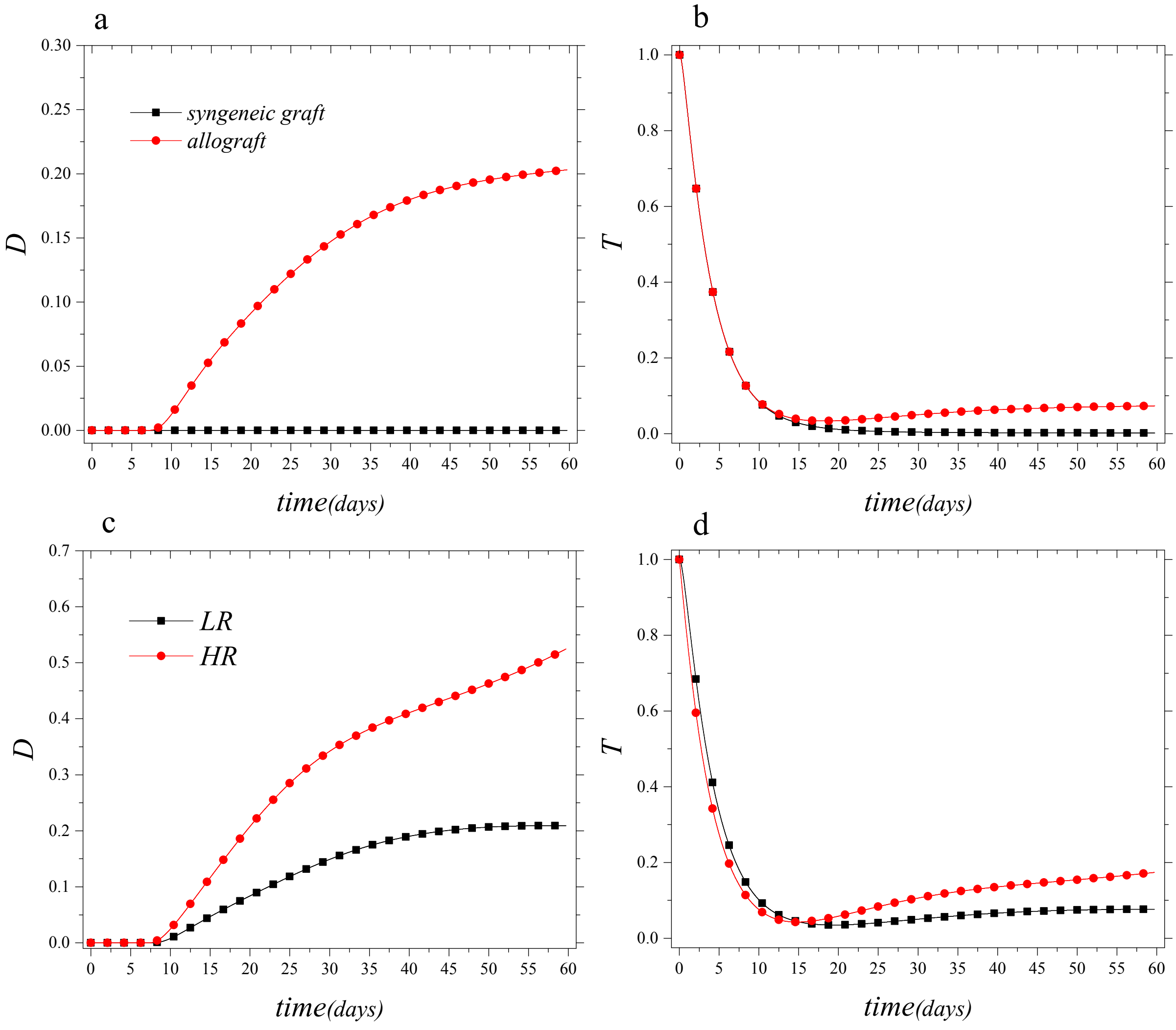

FIG. 3: Dynamics of T and D for syngeneic graft and allograft(a and b). Dynamics of T and D for LR settings and HR settings (c and d).

we calculated the durations of therapy (see Figures S5 and S6). Note that contrary to Figure 9 of the article, here we do not ensure that after cessation of therapy edema score remains below two and therapy has not been administrated after 60 *days*.

$$\frac{dT(t)}{dt} = -\alpha_{V_B T}\frac{V_B(t)}{1+V_B(t)}T(t) + \alpha_{DT}D(t) \quad (1)$$

$$\frac{dV_{BN}(t)}{dt} = -\lambda_{VB}V_{BN}(t) + [\alpha_{TV_{BN}}T(t) + \alpha_{DV_{BN}}D(t)][1 - V_B(t)/V_{Bmax}] \quad (2)$$

$$\frac{dV_L(t)}{dt} = -\lambda_{VL}V_L(t) + [\alpha_{TV_L}T(t) + \alpha_{DV_L}D(t)][1 - V_L(t)/V_{Lmax}] \quad (3)$$

$$\frac{dD_p(t)}{dt} = -\lambda_D D_p(t) + \alpha_{TV_L D_p}T(t-t_d)V_L(t-t_d) \quad (4)$$

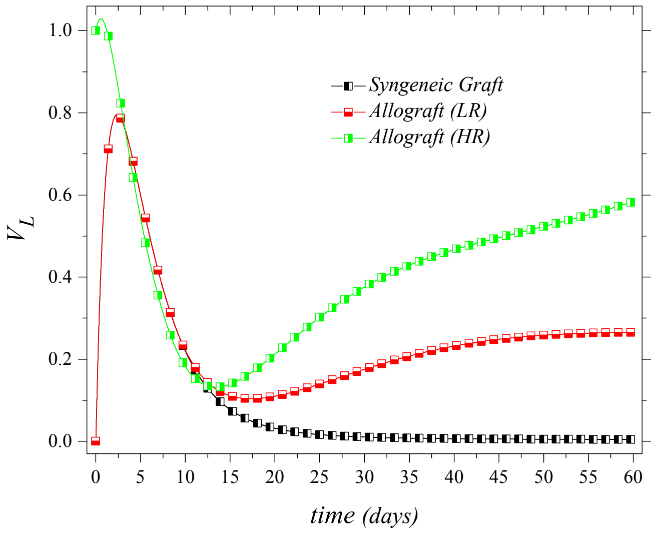

FIG. 4: Evolution of V_L for different graft conditions.

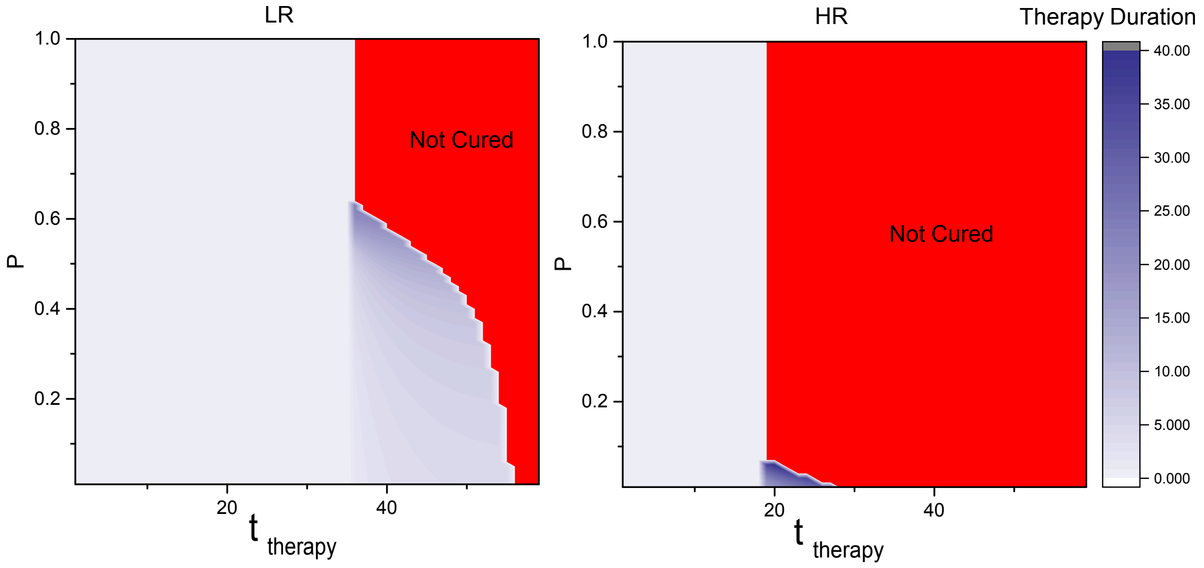

FIG. 5: Duration of therapy for non-specific approach versus the starting time of drug administration and blood (lymphatic) vessel growth suppression factor P $(1-P)$. Please see Figure 3 of the article for temporal variation of edema scores in untreated LR and HR models.

$$\frac{dD(t)}{dt} = -\lambda_D D(t) + \alpha_{V_B D_p D} D_p(t) V_B(t) \tag{5}$$

$$\frac{dE(t)}{dt} = -\lambda_E E(t) - \alpha_{V_L E} V_L(t) + [\alpha_{DV_B E} D(t) V_B(t) + \alpha_{TE} T(t)][1 - E(t)/E_{max}] \tag{6}$$

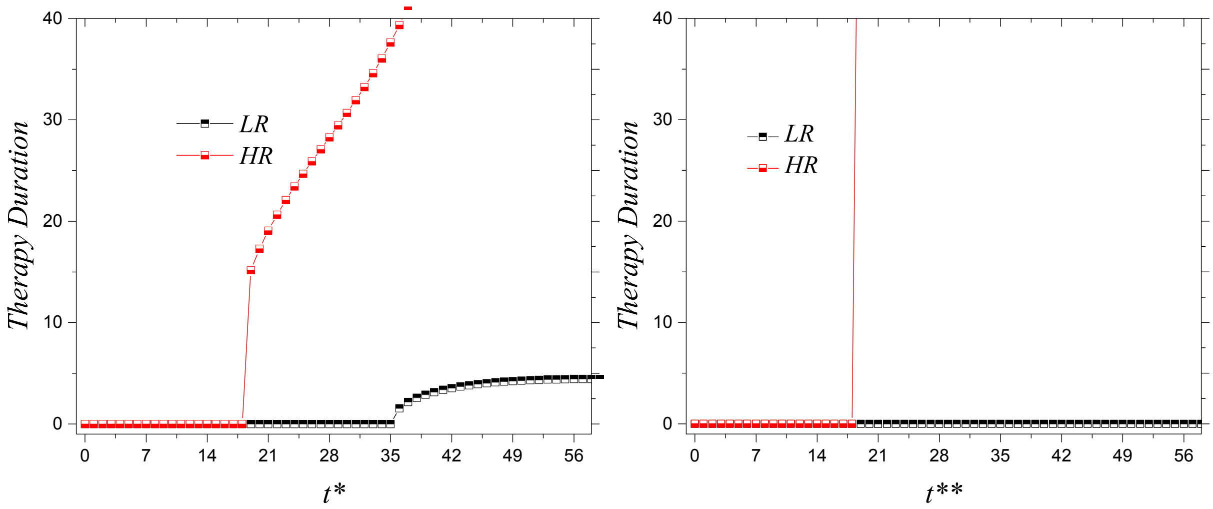

FIG. 6: Duration of therapy for specific approach in HR and LR settings versus specific drug administration time for blood (lymphatic) vessels t^* (t^{**}). Please see Figure 3 of the article for evolution of edema scores in untreated LR and HR models.

* Corresponding Author: a.mashaghi.tabari@lacdr.leidenuniv.nl

[1] T. Inomata, A. Mashaghi, A. Di Zazzo, S.-M. Lee, H. Chiang, and R. Dana, Cornea **36**, 491 (2017).
[2] W. Press, S. Teukolsky, W. Vetterling, and B. Flannery, Numerical Recipes: The Art of Scientific Computing (1986).

TABLE II: Values of coefficients and constants used in the model and their definition.

Coefficient, Constant	definition	Value	**confidence region**
$\alpha_{V_B T}$	The contribution of blood vessels to trauma healing	0.333	0.013
α_{DT}	The contribution of the immune system to tissue damage	0.1	0.02
α_{TE}	The impact of trauma on accumulation of edema within the tissue	2	1.2
$\alpha_{V_L E}$	Tissue edema resolution by lymphatic vessels	0.1	0.4
$\alpha_{D_P V_B D}$	Immune cell trafficking by blood vessels	0.052	0.004
$\alpha_{DV_{BN}}$	New blood vessels formation stimulated by the immune cells	7	0.7
α_{DV_L}	New lymphatic vessel formation stimulated by the immune cells	0.5	1.5
$\alpha_{TV_{BN}}$	New blood vessel formation stimulated by the graft	3	1.4
α_{TV_L}	New lymphatic vessel formation stimulated by the graft	1.95	0.75
α_{DE}	Accumulation of edema within the tissue stimulated by immune cells	1.35	0.5
$\alpha_{TV_L D_P}$	Alloantigen transportation to lymph nodes from graft site by lymphatic vessels.	0.052	0.0045
λ_{VB}	Blood vessels' spontaneous decay	0.16	0.015
λ_{VL}	Lymphatic vessels' spontaneous decay	0.63	0.09
λ_D	Immune cells' spontaneous decay	0.038	0.0045
λ_E	Spontaneous resolution of edema	0.28	0.09
T_{delay}	The time which takes for the immune cells to be present at the graft site	7.4	1.4
V_{Lmax}	The maximum value for lymphatic vessels (capacity of the tissue for lymphatic vessels)	4.6	2.
V_{Bmax}	The maximum value for blood vessels (capacity of the tissue for blood vessels)	8	0.8
E_{max}	The maximum value for edema (capacity of the tissue for edema)	5.35	2.2
